# Preoperative CT Nodal Staging Versus Pathology in Colon Cancer: A Retrospective Multicentre Diagnostic Accuracy Study

**DOI:** 10.3390/cancers18193175

**Published:** 2026-10-01

**Authors:** Tomasz Cwaliński, Mateusz Jagielski, Michał Kazanowski, Jacek Piątkowski, Maciej Ciesielski, Marcin Januszkiewicz, Zuzanna Bigus, Patryk Kaczor, Bartosz Kapturkiewicz, Paweł Lesiak, Sergii Girnyi, Damian Nowiński, Piotr Kurek, Natale Calomino, Marek Jackowski, Marek Bębenek, Luigi Marano

**Affiliations:** 1 Department of General Surgery and Surgical Oncology, Saint Wojciech Hospital, Nicolaus Copernicus Health Center, 80-462 Gdańsk, Poland; tcwalinski@copernicus.gda.pl (T.C.); zbigus@copernicus.gda.pl (Z.B.); sgirnyi@copernicus.gda.pl (S.G.); 2 Department of General, Gastroenterological and Oncological Surgery, Collegium Medicum, Nicolaus Copernicus University in Toruń, 85-067 Bydgoszcz, Poland; matjagiel@gmail.com (M.J.); jpiatkowski@wp.pl (J.P.); patryk.kaczor97@gmail.com (P.K.); jackowscy@hotmail.com (M.J.); 3 Department of Oncology and Haematology, Faculty of Medicine, Wroclaw University of Science and Technology, 50-370 Wroclaw, Poland; michal.kazanowski@dcopih.pl (M.K.); bartosz.kapturkiewicz@dcopih.pl (B.K.); marek.bebenek@dcopih.pl (M.B.); 4 First Department of Surgical Oncology, Lower Silesian Oncology, Pulmonology and Hematology Center, Pl. Hirszfelda 12, 53-413 Wroclaw, Poland; pawel.lesiak@dcopih.pl; 5 Department of Oncological Surgery, Gdynia Oncology Centre, 81-519 Gdynia, Poland; maccies@gumed.edu.pl (M.C.); piotr_kurek@gumed.edu.pl (P.K.); 6 Department of Oncological Surgery, Faculty of Health Sciences with the Institute of Maritime and Tropical Medicine, Medical University of Gdańsk, 81-519 Gdynia, Poland; 7 Department of Minimally Invasive and Oncological General Surgery, Voivodeship Hospital in Bialystok, 15-950 Bialystok, Poland; marcinj74@gmail.com (M.J.); damianboxlek@gmail.com (D.N.); 8 Kidney Transplant Unit, Department of Medicine Surgery and Neuroscience, University Hospital of Siena, 53100 Siena, Italy; natale.calomino@unisi.it; 9 Department of Medicine, Academy of Applied Medical and Social Sciences, AMiSNS, 82-300 Elblag, Poland

**Keywords:** colon cancer, computed tomography, preoperative staging, pathological staging, lymph nodes, diagnostic accuracy, multicentre study

## Abstract

Background/Objectives: Computed tomography (CT) informs preoperative staging in colon cancer, yet its diagnostic accuracy for lymph node involvement relative to pathological staging remains uncertain. We examined nodal concordance and multicentre variation in Poland. Methods: This retrospective diagnostic accuracy study included adults who underwent upfront resection for nonmetastatic colon cancer at five centres between January 2022 and August 2025. All underwent preoperative CT; recorded cN categories were dichotomised as cN0 versus cN+ (any positive cN category), and pathological pN was the reference. We estimated accuracy, sensitivity, specificity, predictive values, and kappa with 95% confidence intervals. A centre-adjusted exploratory analysis examined the association between cT3–4 and occult pN+ among cN0 patients. Results: Of 662 otherwise eligible patients, 590 had evaluable cN and pN. CT and pathology agreed in 386 (65.4%; 95% CI, 61.5–69.2; κ = 0.276; 95% CI, 0.199–0.353). Occult pN+ occurred in 73 of 335 cN0 patients (21.8%; 95% CI, 17.7–26.5); 131 of 255 cN+ patients were pN0 (51.4%; 95% CI, 45.3–57.4). Sensitivity was 62.9% and specificity 66.7%. Centre-level cN/pN completeness ranged from 73.5% to 100%. cT3–4 was associated with occult pN+ after centre adjustment (OR, 3.10; 95% CI, 1.71–5.62). Sensitivity analyses were materially consistent. Conclusions: Routine CT nodal classification showed limited concordance with pathology. The cT3–4 association is exploratory and does not establish a prediction or treatment selection rule; cN alone should not determine treatment.

## Simple Summary

Computed tomography is used before colon cancer surgery to assess whether cancer has spread to nearby lymph nodes. This information may influence treatment discussions, but scan findings do not always match examination of the removed tissue. We compared routine scan-based nodal classifications with surgical pathology in 590 patients treated at five Polish centres. About one in five patients whose scans suggested no nodal involvement had cancer-positive nodes at pathology, while about half of those classified as node-positive on scans had no nodal metastases identified after surgery. More advanced primary tumours were associated with hidden nodal disease among patients classified as node-negative. These findings show why lymph node findings on scans should be considered together with other tumour and patient characteristics during multidisciplinary planning. They do not establish a rule for selecting preoperative treatment.

## 1. Introduction

Preoperative computed tomography (CT) informs both staging and treatment planning in colon cancer. Beyond defining distant disease and local resectability, it increasingly informs treatment sequence. In FOxTROT, CT-derived cT3–4, cN0–2, cM0 disease defined the population considered for perioperative chemotherapy [1,2]. Earlier multicentre work established the feasibility of CT-based selection of locally advanced tumours for neoadjuvant trials [3]. Contemporary European and North American guidance places cross-sectional staging within multidisciplinary treatment planning, while emphasising that imaging findings must be interpreted alongside resectability, tumour biology, and patient fitness [4,5,6]. A category assigned before surgery may thus influence whether a patient proceeds directly to resection or is discussed for preoperative systemic treatment.

The performance of CT is not uniform across staging domains. Earlier systematic reviews consistently identified limitations in nodal assessment despite better performance for local tumour extension or distant disease [7,8,9]. A 2026 meta-analysis of 32 studies and 222,948 patients estimated sensitivity of 0.81 and specificity of 0.75 for identifying pT3–4 disease, but could not derive a reliable pooled estimate for nodal status [10]. A nodal-staging meta-analysis of 29 studies and 5634 patients found pooled sensitivity of 0.693 and specificity of 0.660, without clear improvement from adding morphology to size criteria [11]. CT may thus identify an advanced primary tumour reasonably well while remaining uncertain about regional nodal metastasis.

Several biological and organisational factors may contribute to this uncertainty. Metastatic deposits can occur in nodes that are neither enlarged nor morphologically conspicuous, whereas reactive or inflammatory nodes may appear suspicious. Studies of nodal morphology have examined internal heterogeneity and border irregularity in addition to size, with promising single-study findings that require wider validation [12]. Routine-practice studies from Sweden, Denmark, the Netherlands, and the United States have reported incomplete staging and only modest agreement between clinical and pathological N categories [13,14,15,16,17,18,19,20,21]. A recent multicentre reader study found only moderate reproducibility for both CT T and N stage, despite structured reassessment [22]. Discordance may therefore enter the pathway through image acquisition, interpretation, reporting, case selection, or pathological verification rather than through a single technical failure.

Evidence on routine CT-based nodal staging from Central and Eastern Europe remains limited, particularly regarding diagnostic completeness across centres. Moreover, cN0/pN+ and cN+/pN0 discordance have distinct clinical implications and should be assessed separately: the former represents occult nodal disease, whereas the latter may reflect radiological over-classification, reactive nodes, or an imperfect pathological reference. Using individual-level data from patients undergoing upfront resection at five Polish centres between January 2022 and August 2025, we compared routine CT-derived nodal status with resection pathology. Our primary objective was to quantify occult nodal disease among patients classified as cN0 and pathological node negativity among those classified as cN+. This study extends existing evidence by combining a contemporary Polish multicentre cohort with bidirectional misclassification analysis, centre-level assessment of staging completeness, and sensitivity analyses addressing lymph node yield, data quality, and cohort eligibility. Secondary objectives were to describe centre-level variation, assess cT–pT agreement, and explore the post hoc association between cT3–4 and occult pN+ among patients classified as cN0.

## 2. Materials and Methods

### 2.1. Study Design, Setting, and Reporting

We performed a retrospective, multicentre diagnostic accuracy analysis of the Polish Analysis of Textbook Outcome in Colorectal Cancer Surgery (Pol-ATOCCS) database. The parent project was originally designed to study Textbook Outcome after colorectal cancer surgery, and a domain-specific analysis of Textbook Outcome failure has been published from the broader cohort [23]. The present CT–pathology question was developed as a scientifically distinct secondary analysis after data collection. Five tertiary centres contributed routinely collected surgical records from January 2022 to August 2025. We treated the cN category available during treatment planning as the index classification and postoperative pN as the reference classification. The report follows STARD 2015 as the primary reporting guideline and STROBE as a complementary cohort guideline [24,25]. No formal sample size calculation was performed for this secondary diagnostic question; we analysed all records meeting the analytic criteria. The original Textbook Outcome sample size calculation was not applicable to diagnostic accuracy and is not presented as justification for this analysis.

### 2.2. Participants

We included adults with a primary colon cancer who underwent resection without neoadjuvant therapy during the study period, had no documented metastatic disease, and had evaluable cN and pN categories. Rectal cancers and the single record classified as both colon and rectum were excluded. For the secondary T-stage analysis, otherwise eligible patients contributed when both cT and pT were evaluable, irrespective of N-stage completeness.

The parent Textbook Outcome protocol specified age ≥ 18 years, ASA physical status < IV, histological confirmation, M0 disease, curative resection, no prior colectomy, and no palliative treatment. All patients in the diagnostic cohort were adults and no R2 resection was recorded. Seven patients in the primary diagnostic cohort had recorded ASA IV status, none had recorded ASA V status, and ASA was missing in twelve patients. The presence of ASA IV records represents a departure from the parent protocol’s stated ASA eligibility criterion; the reason for their inclusion in the source database could not be established from the available analytic documentation. These seven records and the twelve records with missing ASA were retained in the present secondary diagnostic analysis because ASA was not used to define either the index classification or the reference classification. A sensitivity analysis requiring age ≥ 18 years, documented ASA < IV, and no R2 resection included 571 patients.

One record coded as ASA V was present among the 72 patients excluded from the primary nodal analysis because of incomplete staging data; this patient had non-evaluable cN. The available analytic data did not permit verification of this ASA V classification against the original anaesthetic record.

Sex was available for every included patient and is reported for the full cohort, with exploratory sex-stratified diagnostic estimates in Supplementary Table S1.

### 2.3. Index Classification: Preoperative CT

Preoperative CT was performed in all included patients. All five participating centres used the same classification system for cT and cN, based on the eighth edition of the AJCC TNM staging system [26]. For this analysis, cN was transcribed directly from the original preoperative CT report and represented the radiologist-assigned nodal classification. Examinations were routinely interpreted by specialist radiologists. The number of imaging assessments or rereadings contributing to the reported cN, and any subsequent modifications during multidisciplinary review, were not documented in the shared database.

All five centres used a common CT reporting template. CT acquisition parameters, including slice thickness, were not captured in the shared database. The nodal size and morphological criteria used to assign cN, together with individual reader characteristics such as years of experience and reporting volume, were also not recorded.

Although the reporting template and AJCC TNM staging framework were shared, uniformity of acquisition protocols and interpretative criteria could not be verified, and the extent of between-centre heterogeneity in these aspects could not be quantified.

For this analysis, recorded cN0 was treated as node-negative and every positive cN category was collapsed into cN+; cN+ is thus an analytic binary grouping rather than a separate AJCC category. Because cN was assigned before operation and pathological examination, the index result necessarily preceded the reference result.

### 2.4. Reference Classification: Resection Pathology

Pathological T and N categories were abstracted from the definitive local resection report and interpreted according to the eighth edition of the AJCC staging system [26]. pN0 was treated as absence of the target condition and any positive pathological nodal category as pN+. Definitive resection pathology was selected because it is the clinical reference for regional nodal status after upfront surgery.

A pN0 classification may be less secure when fewer than 12 nodes are examined; nodal yield is therefore relevant to the completeness of pathological staging [5,27]. We therefore performed two reference-standard stress tests: one restricted all patients to a yield of at least 12 nodes, and the other retained every pN+ case but required at least 12 examined nodes for pN0. The latter changes the analysed disease spectrum and was interpreted as a sensitivity analysis, not as an alternative prevalence estimate.

### 2.5. Outcomes and Data Harmonisation

The co-primary descriptive outcomes were occult nodal disease among patients classified as cN0 (cN0/pN+) and cN+/pN0 discordance. We also estimated overall accuracy, sensitivity and specificity for pN+, positive and negative predictive values, and Cohen’s κ. Secondary outcomes included centre-specific cN/pN completeness and occult pN+ rates, tumour-side-stratified diagnostic estimates, exact cT–pT agreement, CT under- and over-classification, and linear-weighted κ for T category.

Clinical and pathological nodal status was harmonised into binary categories (cN0 versus cN+ and pN0 versus pN+, respectively), using the dedicated cN and pN variables for the primary analysis. The source data dictionary defined cN code 1 as N+, rather than specifically N1. A consistent distinction between cN1 and cN2 could not therefore be established across the cohort from the available database fields. Positive clinical nodal codes were consequently harmonised as cN+ rather than interpreted as uniform AJCC N1 or N2 categories. Data quality checks identified five anomaly flags involving four distinct patient records: one out-of-range cN entry, one out-of-range pN entry, and three disagreements between the dedicated pN field and the derived AJCC stage group. The out-of-range pN entry occurred in one of the three records with a pN/stage-group disagreement. Sensitivity analyses excluded either the two records with out-of-range nodal entries (N = 588) or all four records with staging-field anomalies (N = 586), with materially similar estimates.

Tumour side was grouped as right/transverse (caecum to transverse colon) or left/rectosigmoid (splenic flexure to rectosigmoid). Unclassifiable records were excluded only from site-specific summaries. Operative procedure categories were clinically reviewed and harmonised (Supplementary Table S5). Records with a repeated source participant identifier were flagged during database preparation. Eight flagged records met the eligibility criteria for the primary analysis. Their clinical data were not identical, and the de-identified analytic extract alone did not permit definitive distinction between separate procedures, duplicate entries, and identifier errors. Flagged records were retained to avoid unverified deletion of potentially valid observations or arbitrary selection of one record. A sensitivity analysis excluded all eight flagged records, leaving 582 observations.

### 2.6. Statistical Analysis

**Figure 1:**
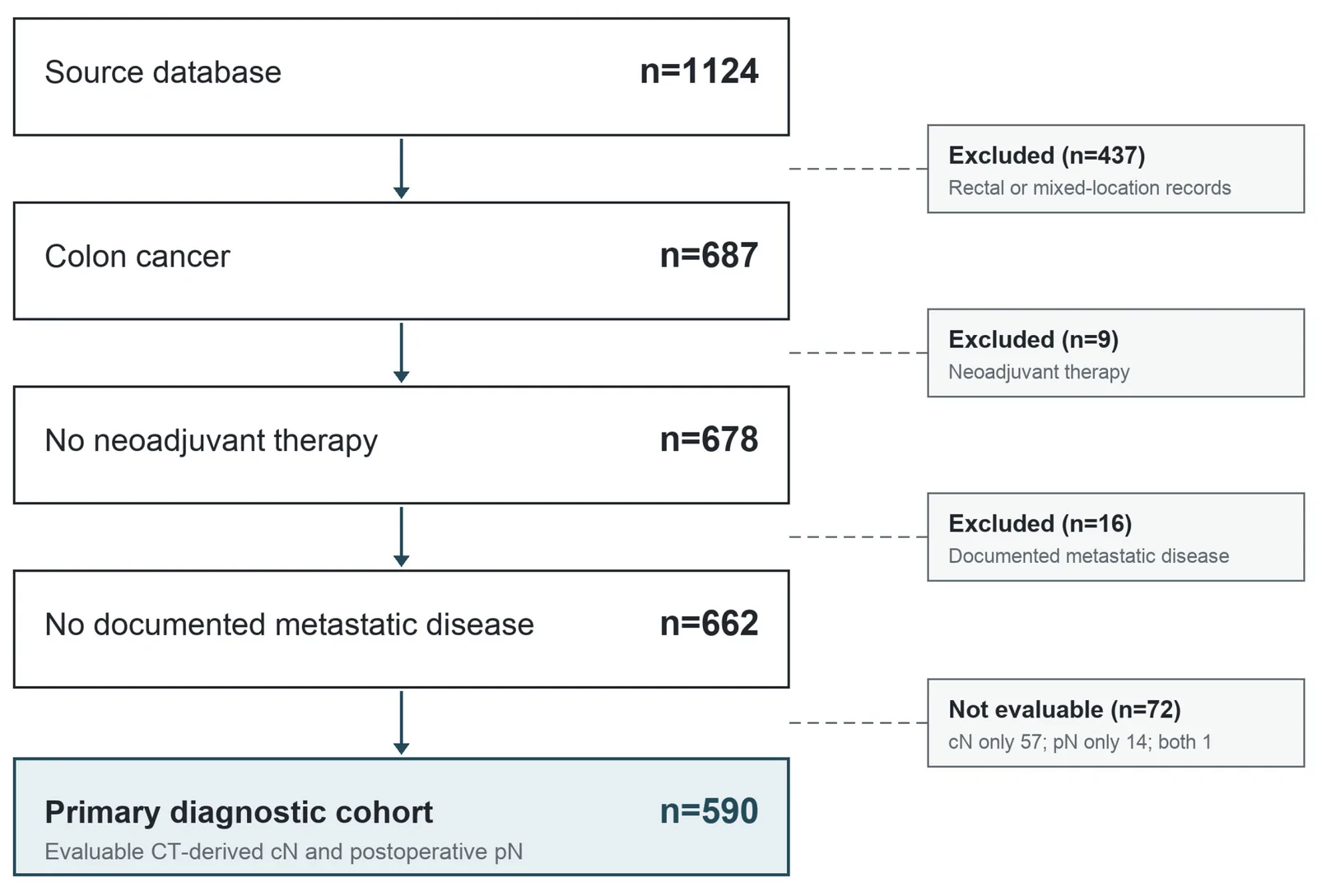
Participant flow for the diagnostic accuracy analysis. The primary cohort comprised 590 patients with evaluable CT-derived clinical nodal category (cN) and postoperative pathological nodal category (pN). Among the 72 otherwise eligible patients excluded from the primary 2 × 2 analysis, 57 lacked evaluable cN only, 14 lacked evaluable pN only, and 1 lacked both categories. All included patients underwent preoperative computed tomography; unavailable cN reflects missing or non-evaluable database staging rather than absence of CT.

Patients with missing or non-evaluable cN or pN did not enter the primary 2 × 2 table. We report the number and pattern of these exclusions overall and by centre, together with descriptive characteristics of included and excluded patients (Figure 1 and Supplementary Tables S3 and S4). No values were imputed. Denominators are shown for variables with missing observations. Descriptive cT denominators include cT0, which is excluded from cT1–2 versus cT3–4 comparisons.

Categorical variables are presented as n/N (%) and continuous variables as median (interquartile range). Wilson 95% confidence intervals (CIs) accompany diagnostic proportions [28]. We calculated Cohen’s κ for binary nodal status and linear-weighted κ for ordered T categories [29,30]; κ CIs were obtained from 5000 nonparametric bootstrap samples using a fixed random seed. Centre-specific proportions were descriptive and were not used to rank centres.

Among cN0 patients, an exploratory complete-case logistic model estimated the association between cT3–4 (versus cT1–2) and occult pN+, with centre entered as a fixed effect. This parsimonious model contained six parameters including the intercept and 73 events. An extended sensitivity model additionally included age per 10 years, sex, and tumour side. We did not use automated variable selection, derive a risk score, or assess a treatment effect. Odds ratios (ORs) are reported with Wald 95% CIs; all tests were two-sided and secondary analyses were not adjusted for multiplicity. Side-stratified estimates and operative characteristics were post hoc and descriptive; no between-group inference was performed.

Sensitivity analyses required ≥12 nodes in all patients, applied the pN0-specific reference-standard restriction, excluded all eight records flagged for possible duplication, excluded the four patient records with staging-field anomalies, and restricted the sample to documentable parent protocol criteria. *p* values below 0.05 were considered statistically significant. Statistical analyses were performed using Stata, version 19.0 (StataCorp LLC, College Station, TX, USA).

### 2.7. Ethics and Data Governance

This study was conducted in accordance with the Declaration of Helsinki and approved by the Bioethics Committee of the Academy of Applied Medical and Social Sciences (AMiSNS), Elbląg, Poland (Resolution KB10/2024; 10 July 2024). The approval covered the retrospective inclusion and analysis of routinely collected clinical data from patients treated before 10 July 2024, as well as data from patients treated after this date within the approved parent study. No study-specific intervention or prospective research enrolment took place before approval. The requirement for individual informed consent was waived by the Bioethics Committee because of the retrospective, non-interventional design and the use of de-identified routinely collected clinical data. No identifiable participant information is reported.

## 3. Results

### 3.1. Cohort Selection and Missingness

The source database contained 1124 records. After restricting to colon cancer (n = 687), no neoadjuvant therapy (n = 678), and no documented metastatic disease (n = 662), 590 patients had evaluable cN and pN and formed the primary diagnostic cohort (Figure 1). Among the 72 otherwise eligible patients excluded from the 2 × 2 analysis, 57 lacked evaluable cN only, 14 lacked evaluable pN only, and 1 lacked both. Missing cN represented an absent or non-evaluable database field, not absence of preoperative CT.

Median age in the diagnostic cohort was 71 years (IQR, 65–77), and 300 patients (50.8%) were female (Table 1). The 72 patients without complete cN/pN were somewhat older (median, 74 years), included fewer women (43.1%), and more often had ASA III or higher (40.3% versus 34.3% among evaluable patients). For cT, 30 of 72 excluded patients and 1 of 590 included patients had missing or unclassifiable values; cT0 was retained in descriptive summaries. Tumour side was classifiable in 586 patients (right/transverse, 288; left/rectosigmoid, 298). The leading operations were right hemicolectomy (259/590, 43.9%), sigmoidectomy (202/590, 34.2%), and left colectomy (96/590, 16.3%); 201 procedures were open, 204 laparoscopic, and 185 robotic (Supplementary Table S5). Diagnostic completeness varied by centre from 73.5% to 100% (Supplementary Tables S3 and S4), making selection related to centre and staging completeness plausible. Among patients with evaluable cT, cT3–4 was recorded in 19/42 excluded patients (45.2%) and 423/589 included patients (71.8%); this comparison is limited by the substantially greater cT missingness among excluded patients. Right/transverse tumours accounted for 31/72 (43.1%) and 288/586 (49.1%), respectively. Of the 72 excluded patients, 36 were from centre B, 35 from centre D, and 1 from centre E. Both groups underwent resection without neoadjuvant therapy, as required by the eligibility criteria (Supplementary Tables S3 and S4).

### 3.2. Nodal Diagnostic Performance

**Figure 2:**
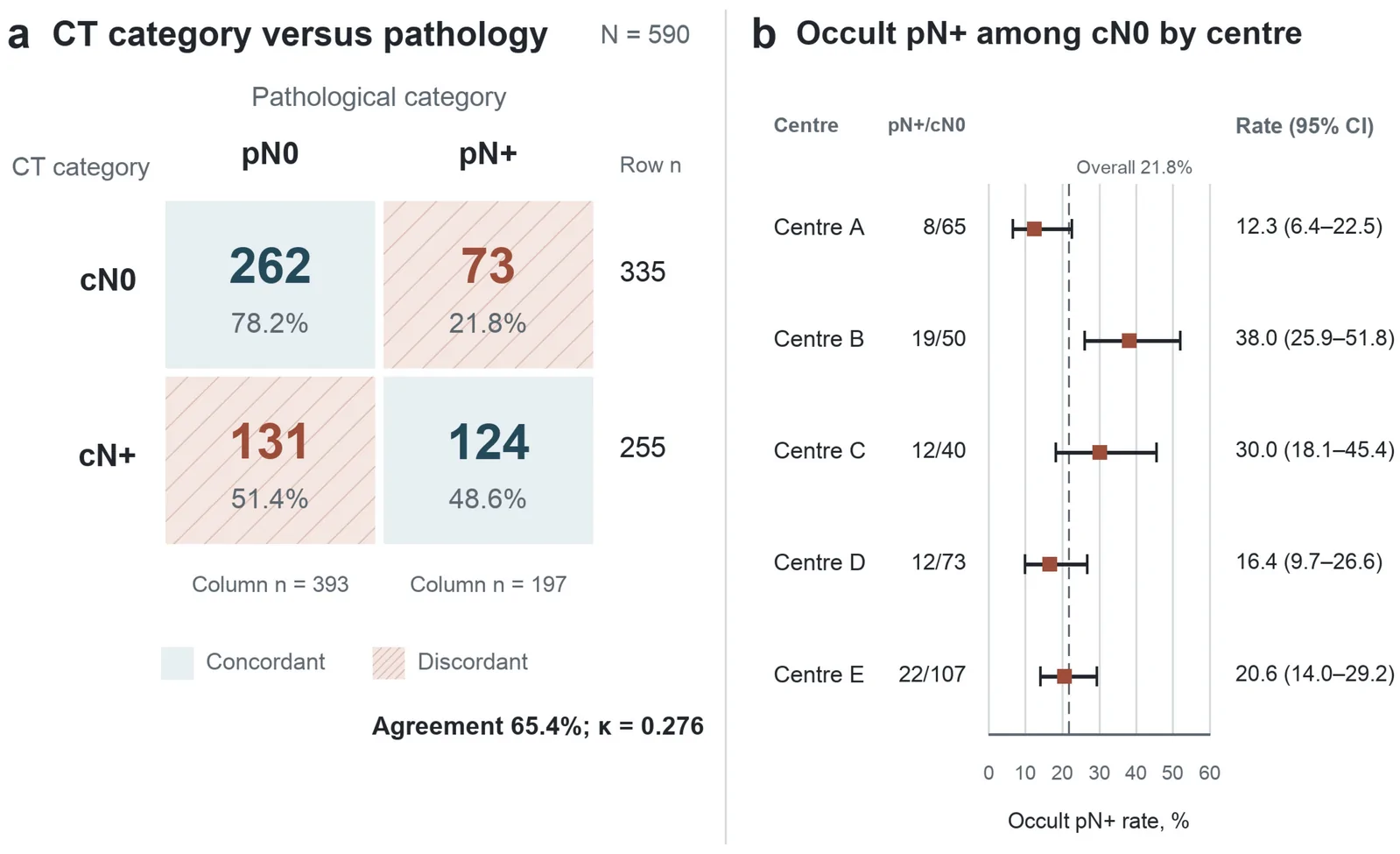
Preoperative CT-derived versus pathological binary nodal status. (**a**) The 2 × 2 distribution comprised 262 cN0/pN0, 73 cN0/pN+, 131 cN+/pN0, and 124 cN+/pN+ cases. Percentages are calculated within each binary cN group; hatched cells represent discordant classifications. Overall agreement was 65.4%, with a Cohen’s κ of 0.276. (**b**) Centre-specific proportions of occult pN+ among patients classified as cN0. Squares indicate point estimates, horizontal lines indicate Wilson 95% confidence intervals, and the dashed vertical line represents the overall occult pN+ rate of 21.8%. Centres are anonymised. Centre-level estimates are descriptive and should not be interpreted as rankings because case mix and completeness of cN and pN data differed. cN+ and pN+ denote analytic groupings of all positive clinical and pathological nodal categories, respectively; neither is a separate AJCC TNM category. CI, confidence interval; cN, clinical nodal category; CT, computed tomography; pN, pathological nodal category.

Binary CT-derived and pathological nodal classifications agreed in 386 of 590 patients (65.4%; 95% CI, 61.5–69.2), with κ = 0.276 (95% CI, 0.199–0.353). The 2 × 2 distribution comprised 262 cN0/pN0, 73 cN0/pN+, 131 cN+/pN0, and 124 cN+/pN+ cases (Figure 2 and Table 2). Sensitivity for pN+ was 62.9% (95% CI, 56.0–69.4), specificity 66.7% (95% CI, 61.9–71.1), positive predictive value 48.6% (95% CI, 42.6–54.7), and negative predictive value 78.2% (95% CI, 73.5–82.3).

Occult pN+ was present in 73 of 335 patients classified as cN0 (21.8%; 95% CI, 17.7–26.5). Conversely, 131 of 255 patients classified as cN+ were pN0 (51.4%; 95% CI, 45.3–57.4). The occult pN+ rate ranged from 12.3% to 38.0% across centres, but these estimates had wide and overlapping CIs and arose in the setting of differential cN/pN completeness; they were not interpreted as centre rankings (Figure 2).

Sex-stratified estimates were similar descriptively. Accuracy was 64.5% (95% CI, 58.8–69.8) in men and 66.3% (95% CI, 60.8–71.4) in women; occult pN+ among cN0 was 22.4% and 21.2%, respectively (Supplementary Table S1). This study was not designed to establish equivalence between sexes.

Sensitivity was 70.0% for right/transverse and 55.8% for left/rectosigmoid tumours; specificity was 66.0% and 67.0%, and occult pN+ among cN0 was 19.5% and 23.6%, respectively. CIs overlapped, and tumour side showed no clear association in the extended centre-adjusted model (OR, 1.04; 95% CI, 0.57–1.92; *p* = 0.895). The restricted side comparison was similar (Supplementary Table S6).

### 3.3. cT Category and Occult pN+

Among cN0 patients with cT1–4, cT3–4 was recorded in 52 of 73 patients with pN+ (71.2%) and 129 of 258 with pN0 (50.0%) (Supplementary Table S7). The centre-adjusted model included 331 patients and 73 events. cT3–4 was associated with higher odds of occult pN+ than cT1–2 (OR, 3.10; 95% CI, 1.71–5.62; *p* < 0.001). The estimate was similar after adding age, sex, and tumour side (OR, 3.19; 95% CI, 1.71–5.94), after excluding the three cN0 patient records with staging-field anomalies (OR, 2.93; 95% CI, 1.58–5.44), and after restricting to documentable protocol criteria (OR, 3.03; 95% CI, 1.64–5.62) (Supplementary Table S8).

### 3.4. T-Stage Agreement

The secondary T-stage analysis was performed in the 662 otherwise eligible patients, independently of cN/pN completeness. Both cT and pT (T0–T4) were evaluable in 618 patients. The remaining 44 patients comprised 29 with non-evaluable cT but evaluable pT, 13 with evaluable cT but non-evaluable pT, and 2 with neither category evaluable. Thus, cT was missing or unclassifiable in 31 patients and pT in 15, with an overlap of 2 patients. Of the 618 patients included in the paired T-stage analysis, 589 belonged to the primary nodal analysis cohort of 590 patients, whereas 29 belonged to the group of 72 patients excluded from the nodal analysis. Categories agreed exactly in 387 (62.6%); CT under-classified T in 76 (12.3%) and over-classified it in 155 (25.1%). Linear-weighted agreement was moderate (κ = 0.502; 95% CI, 0.448–0.554) (Supplementary Table S9).

### 3.5. Sensitivity Analyses

Discordance did not diminish in the 462 patients with at least 12 nodes examined: occult pN+ occurred in 58 of 239 cN0 patients (24.3%; 95% CI, 19.3–30.1), and 122 of 223 cN+ patients were pN0 (54.7%; 95% CI, 48.2–61.1). Nodal yield completeness within each cN/pN cell is shown in Supplementary Table S10. When all pN+ cases were retained but pN0 required at least 12 nodes, the occult pN+ proportion was 73 of 254 (28.7%; 95% CI, 23.5–34.6); this stress test changes the analysed spectrum and should not be read as a replacement prevalence estimate.

The occult pN+ estimate was 21.5% after excluding all eight records flagged for possible duplication, 21.1% after excluding the four patient records with staging-field anomalies, and 22.0% after restricting to documentable parent protocol criteria. Corresponding κ estimates remained between 0.212 and 0.286 (Table 3 and Supplementary Table S2).

## 4. Discussion

This study evaluates CT-derived nodal classification as documented in routine clinical radiology reports; its diagnostic estimates should not be interpreted as the performance of CT under a verified, standardised acquisition and interpretation protocol with central expert review. In this contemporary five-centre cohort, binary CT-derived and pathological nodal classifications disagreed in 204 of 590 patients (34.6%). The discordance was bidirectional: 21.8% of cN0 patients were pN+, whereas 51.4% of cN+ patients were pN0. These patterns have different possible explanations. cN0/pN+ may reflect nodal metastases below the resolution or interpretive threshold of CT. cN+/pN0 may reflect reactive nodes or radiological over-classification, although incomplete pathological detection remains possible when nodal yield is limited.

The magnitude of disagreement is not unique to this cohort. Our sensitivity of 62.9% and specificity of 66.7% are close to the pooled estimates of 69.3% and 66.0%, respectively, reported in the recent 29-study nodal meta-analysis [11]. A Swedish population study reported κ = 0.28 for cN versus pN, closely resembling the present estimate [13]. A later Swedish analysis found that 52% of patients classified as cN+ had pN0 disease [21]. Dutch nationwide data likewise showed frequent under- and over-classification and considerable between-hospital variation in staging completeness [20]. These comparisons are contextual rather than benchmark values: predictive values and agreement depend on disease prevalence, case mix, thresholds, missingness, and the reference standard.

The clinical relevance lies less in any single percentage than in the uncertainty surrounding a binary cN label. FOxTROT demonstrated that preoperative chemotherapy can improve selected pathological and disease control outcomes in radiologically staged locally advanced colon cancer [1]. Our cohort excluded neoadjuvant treatment and cannot estimate its benefit or identify who should receive it, nor can a pN0 resection be interpreted as evidence that preoperative treatment would have been inappropriate. The data instead indicate that cN should be integrated with primary-tumour features, resectability, nodal morphology where available, molecular findings, patient fitness, and multidisciplinary judgement. The pathological responses reported with neoadjuvant immunotherapy in mismatch repair-deficient colon cancer further illustrate why molecular findings need to accompany imaging in treatment discussions [31].

cT3–4 was associated with approximately threefold higher odds of occult pN+ after centre adjustment, and the estimate changed little in several sensitivity models. The direction is clinically plausible because deeper local invasion accompanies a higher prior probability of lymphatic spread. Its magnitude remains uncertain. The analysis was post hoc, contained 73 events, and did not include image-level features such as nodal morphology or extramural vascular invasion. Both cT and cN were derived from the same routine CT staging process and may therefore share acquisition-related and interpretive sources of error. The cT3–4 finding represents an exploratory association within the cN0 subgroup and should not be interpreted as evidence that cT provides an independent diagnostic test for nodal involvement. It does not establish a validated prediction rule or treatment selection criterion.

Sensitivity appeared lower for left/rectosigmoid tumours, but CIs overlapped and the adjusted model showed no clear side association. This post hoc pattern could reflect anatomy, cT distribution, reader thresholds, centre case mix, or chance and should not define a side-specific CT rule.

Centre-level occult pN+ proportions ranged from 12.3% to 38.0%, while staging completeness varied from 73.5% to 100%. Harmonisation to cN0 versus cN+ cannot remove variation in underlying image-level criteria. Previous CT studies have evaluated short-axis size thresholds ranging from 4 to 10 mm and morphological features such as internal heterogeneity and irregular outer borders [12]. These criteria provide relevant methodological context, but the specific thresholds and morphological combinations underlying the recorded cN classifications could not be verified in our cohort because they were not captured in the shared database.

Differences could reflect acquisition, reader practice, documentation, referral patterns, tumour biology, pathology, or chance. With only five centres and incomplete workflow data, performance ranking would be unjustified. A prospective audit would be more informative if it recorded scan parameters, explicit size and morphological criteria, reader experience, structured reporting, CT-to-surgery interval, multidisciplinary changes, and blinded central review. The only moderate interobserver reproducibility observed in a recent multicentre reader study reinforces the need to separate acquisition, interpretation, and reporting effects [22]. Training and practice can improve T staging while N staging remains less reliable, supporting separate quality assessment of the two domains [32].

Pathology is the practical postoperative reference but is not infallible. Restricting all patients to at least 12 examined nodes did not reduce discordance, which argues against low yield as its sole explanation. The pN0-specific stress test produced a higher occult proportion because it selectively removed negative references with limited nodal assessment; that result illustrates the direction of possible reference-standard bias rather than a corrected disease frequency. Excluding the four patient records with staging-field anomalies had little effect on the diagnostic estimates.

Prevention and early detection complement accurate staging within the colorectal cancer care pathway. Healthcare staff should also be encouraged to participate in screening according to established age- and risk-based recommendations [33]. In an Austrian workplace screening initiative, 2390 of 10,239 eligible healthcare employees (23.3%) participated in stool-based screening despite facilitated access, illustrating that employment in healthcare does not ensure participation [34]. Workplace initiatives should therefore address participation barriers and facilitate access to recommended screening tests and timely colonoscopy following a positive stool test [33,34]. These preventive measures complement the diagnostic pathway but were not evaluated in the present study, which focused on CT–pathology concordance after cancer diagnosis.

Artificial intelligence-assisted colonoscopy offers a complementary approach to improving colorectal lesion detection. Real-time computer-aided detection (CADe) systems highlight suspected mucosal lesions to support endoscopists during inspection. In a multicentre randomised trial, Repici et al. reported a higher adenoma detection rate with CADe than with conventional colonoscopy (54.8% versus 40.4%) [35]. However, a subsequent international randomised trial found no statistically significant increase in adenoma detection, illustrating variability in clinical effectiveness [36]. These findings support evaluating CADe within specific endoscopy settings while maintaining high-quality mucosal inspection. The cited trials assessed detection outcomes and do not establish a reduction in colorectal cancer mortality. Furthermore, CADe addresses mucosal lesion detection rather than regional nodal staging, and its potential benefits cannot be extrapolated to the CT–pathology discordance examined in the present study.

Deep learning is also being investigated for tissue classification and diagnostic support in colorectal histopathology. Convolutional neural networks can distinguish tumour epithelium, normal mucosa, stroma, and other tissue components in haematoxylin-and-eosin-stained images, enabling quantitative assessment of tissue composition [37]. Multicentre research has also evaluated colorectal cancer recognition in whole-slide images using semi-supervised learning, which combines labelled and unlabelled data to reduce manual annotation requirements [38]. Such approaches may support tumour localisation and pathological review, but clinical implementation requires validation for the intended task across laboratories and routine workflows. Performance in tissue classification or primary-tumour recognition does not itself establish accuracy for identifying nodal metastases or assigning pN. The present study used routinely reported resection pathology as its reference and did not evaluate digital slide analysis or AI-assisted pathological assessment.

Several limitations deserve emphasis. This retrospective secondary analysis was restricted to patients with nonmetastatic colon cancer who underwent upfront resection and had evaluable cN and pN data. It therefore does not represent the full population of patients considered to have potentially resectable colon cancer at diagnosis. Selection for upfront surgery, together with the requirement for evaluable staging data, may have altered the patient and tumour spectrum and influenced the observed diagnostic estimates. Generalisation to patients selected for neoadjuvant therapy or managed without resection requires separate evaluation. cN and/or pN was unavailable for 72 of 662 otherwise eligible patients (10.9%). Staging completeness ranged from 73.5% to 100% across centres, with 71 of the 72 exclusions occurring in centres B and D. Excluded patients were descriptively older, more often had ASA III–V, and had substantially more missing or unclassifiable cT values (Supplementary Tables S3 and S4). Restricting the analysis to evaluable cN/pN pairs may therefore have altered both patient case mix and centre representation, potentially biasing diagnostic estimates and limiting their generalisability to the full eligible cohort. The direction and magnitude of this bias cannot be established from the available data, and the sensitivity analyses addressing nodal yield and data quality do not directly address this source of bias. The Wilson confidence intervals for pooled diagnostic proportions quantify patient-level uncertainty and do not account for potential within-centre correlation arising from shared imaging and reporting practices. If such correlation is present, these intervals may underestimate uncertainty. The estimates should therefore be interpreted as descriptive results from the participating five-centre cohort, with caution when extrapolating to other settings.

Potentially relevant biological variables such as mismatch repair status were unavailable, and treatment or survival consequences of discordance were not examined. CT acquisition parameters, image-level positivity criteria, reader experience, the CT-to-surgery interval, and pathological processing details were not captured in the shared database. Central image reassessment and blinding of pathological assessment to the CT report could not be evaluated. Binary nodal classification also entails a loss of information about the extent of nodal disease. Although appropriate for assessing discordance in the presence or absence of nodal involvement, it does not capture differences between N1 and N2 or discrepancies within the node-positive group. Our diagnostic estimates should therefore not be interpreted as measures of agreement on nodal burden. A category-level analysis would require verification and harmonisation of the individual clinical and pathological N categories against the original reports.

Against these constraints, this study provides individual-level estimates from a region with limited published data, separates the two clinically different directions of N-stage discordance, reports differential missingness, and tests the findings against nodal yield and data quality assumptions. Routine CT cN status may be useful as one probabilistic component of preoperative assessment, but the present data do not support using it as a definitive threshold to trigger or withhold treatment.

## 5. Conclusions

In patients with nonmetastatic colon cancer who underwent upfront resection and had evaluable cN and pN data, routine CT-derived binary nodal classification showed limited concordance with resection pathology, with clinically relevant discordance in both directions. cT3–4 was associated with occult pN+ among patients classified as cN0, but this exploratory association does not establish a treatment selection rule. CT-derived cN should be interpreted alongside primary-tumour features, molecular findings, patient fitness, and multidisciplinary assessment. Prospective studies with standardised imaging and pathological assessment are needed to clarify how these findings can inform treatment planning.

## Figures and Tables

**Table 1:** Characteristics of the primary diagnostic cohort.

Characteristic	Overall (N = 590)	cN0 (n = 335)	cN+ (n = 255)
Age, years, median (IQR)	71.0 (65.0–77.0)	72.0 (65.0–77.0)	71.0 (65.0–76.0)
Female sex, n/N (%)	300/590 (50.8%)	165/335 (49.3%)	135/255 (52.9%)
BMI, kg/m^2^, median (IQR)	26.6 (23.8–29.7)	26.7 (24.2–30.0)	26.5 (23.0–29.7)
ASA III–IV, n/N (%)	198/578 (34.3%)	116/331 (35.0%)	82/247 (33.2%)
Right/transverse colon, n/N (%)	288/586 (49.1%)	154/332 (46.4%)	134/254 (52.8%)
Left/rectosigmoid colon, n/N (%)	298/586 (50.9%)	178/332 (53.6%)	120/254 (47.2%)
cT3–4, n/N (%)	423/589 (71.8%)	181/334 (54.2%)	242/255 (94.9%)
At least 12 nodes examined, n/N (%)	462/532 (86.8%)	239/293 (81.6%)	223/239 (93.3%)
Nodes examined, median (IQR)	18 (14–23)	16 (13–21)	19 (16–26)
pN+, n/N (%)	197/590 (33.4%)	73/335 (21.8%)	124/255 (48.6%)
R0 resection, n/N (%)	579/584 (99.1%)	332/332 (100.0%)	247/252 (98.0%)

Data are median (IQR) or n/N (%). Denominators vary because missing values were not imputed. The cT denominator includes cT0. cN+ and pN+ denote analytic groupings of all positive clinical and pathological nodal categories, respectively; neither is a separate AJCC TNM category. ASA, American Society of Anesthesiologists physical status; BMI, body mass index; cN, CT-derived clinical nodal category; cT, CT-derived clinical tumour category; IQR, interquartile range; R0, microscopically margin-negative resection.

**Table 2:** Comparison of binary CT-derived and pathological nodal classifications.

Measure	n/N	Estimate	95% CI
cN0/pN0	262/590	44.4%	—
cN0/pN+	73/590	12.4%	—
cN+/pN0	131/590	22.2%	—
cN+/pN+	124/590	21.0%	—
Accuracy	386/590	65.4%	61.5–69.2%
Sensitivity for pN+	124/197	62.9%	56.0–69.4%
Specificity for pN0	262/393	66.7%	61.9–71.1%
Positive predictive value	124/255	48.6%	42.6–54.7%
Negative predictive value	262/335	78.2%	73.5–82.3%
Occult pN+ among cN0	73/335	21.8%	17.7–26.5%
pN0 among cN+	131/255	51.4%	45.3–57.4%
Cohen’s κ	N = 590	0.276	0.199–0.353

Pathological pN was the reference classification. Sensitivity is the proportion of pN+ classified as cN+; specificity is the proportion of pN0 classified as cN0. Proportion CIs are Wilson intervals; the κ CI is a percentile interval from 5000 nonparametric bootstrap samples. cN+ and pN+ denote analytic groupings of all positive clinical and pathological nodal categories, respectively; neither is a separate AJCC TNM category. CI, confidence interval; cN, CT-derived clinical nodal category; CT, computed tomography; pN, pathological nodal category.

**Table 3:** Sensitivity analyses for nodal discordance.

Analysis	N	cN0/pN+, n/N (%)	cN+/pN0, n/N (%)	κ (95% CI)
Primary	590	73/335 (21.8%)	131/255 (51.4%)	0.276 (0.199–0.353)
All patients with ≥12 nodes	462	58/239 (24.3%)	122/223 (54.7%)	0.212 (0.125–0.298)
pN0 requires ≥12 nodes	500	73/254 (28.7%)	122/246 (49.6%)	0.217 (0.134–0.300)
Exclude eight records flagged for possible duplication	582	71/330 (21.5%)	130/252 (51.6%)	0.277 (0.196–0.352)
Exclude four records with staging-field anomalies	586	70/332 (21.1%)	130/254 (51.2%)	0.285 (0.208–0.361)
Documentable parent protocol criteria	571	72/328 (22.0%)	122/243 (50.2%)	0.286 (0.208–0.362)

The pN0-specific ≥ 12-node analysis selectively changes the negative reference set and is a stress test rather than a replacement prevalence estimate. Parent protocol sensitivity required age ≥ 18 years, documented ASA < IV, and no R2 resection. cN+ and pN+ denote analytic groupings of all positive clinical and pathological nodal categories, respectively; neither is a separate AJCC TNM category. ASA, American Society of Anesthesiologists physical status; CI, confidence interval; cN, CT-derived clinical nodal category; pN, pathological nodal category; R2, macroscopically incomplete resection.

## Data Availability

The de-identified participant-level dataset is not publicly deposited because it derives from a multicentre clinical collaboration and remains subject to institutional and ethics permissions. A data dictionary, analytic definitions, and reproducible code may be shared on reasonable request to the corresponding author, subject to approval by the ATOCCS Poland steering group and participating institutions.

## Supplementary Materials

The following supporting information can be downloaded at https://www.mdpi.com/article/10.3390/cancers18193175/s1: Table S1: Exploratory sex-stratified diagnostic estimates; Table S2: Complete diagnostic sensitivity analyses; Table S3: Characteristics of patients with and without evaluable cN/pN; Table S4: Diagnostic completeness and occult pN+ by anonymised centre; Table S5: Tumour location, corrected operative procedure, and surgical approach; Table S6: Post hoc diagnostic estimates by clinical tumour side; Table S7: Characteristics of cN0 patients by pathological nodal status; Table S8: Exploratory logistic models for occult pN+ among cN0 patients; Table S9: Cross-classification of CT and pathological T category; Table S10: Nodal yield adequacy across cN/pN cells. Completed STARD 2015 and STROBE reporting checklists are provided separately.
