# Supplementary material for: Preoperative CT Nodal Staging Versus Pathology in Colon Cancer: A Retrospective Multicentre Diagnostic Accuracy Study
Source: Cancers (Basel). 2026 Oct 1;18(19):3175. doi: 10.3390/cancers18193175 (PMC13664425; doi:10.3390/cancers18193175)
Supplement: Supplementary file 1 [file cancers-18-03175-s001.zip › cancers-4590896-supplementary.pdf]

Supplementary Material

Preoperative CT Nodal Staging Versus Pathology in Colon Cancer: A Retrospective Multicentre Diagnostic Accuracy Study

Table S1. Exploratory sex-stratified diagnostic estimates.

Sex	N	cN0/pN0; cN0/pN+; cN+/pN0; cN+/pN+	Accuracy (95% CI)	Sensitivity (95% CI)	Specificity (95% CI)	Occult pN+ among cN0 (95% CI)
**Male**	290	132/38/65/55	64.5% (58.8–69.8)	59.1% (49.0–68.6)	67.0% (60.2–73.2)	22.4% (16.7–29.2)
**Female**	300	130/35/66/69	66.3% (60.8–71.4)	66.3% (56.8–74.7)	66.3% (59.5–72.6)	21.2% (15.7–28.1)

Sex was recorded as male/female in the source database; gender identity was not collected. These descriptive analyses were required for sex-based reporting and were not designed as equivalence or interaction tests. CI, confidence interval; cN, CT-derived clinical nodal category; pN, pathological nodal category.

Table S2. Complete diagnostic sensitivity analyses.

Analysis	N	Accuracy (95% CI)	Sensitivity (95% CI)	Specificity (95% CI)	cN0/pN+ (95% CI)	cN+/pN0 (95% CI)	κ (95% CI)
**Primary**	590	386/590; 65.4% (61.5–69.2)	124/197; 62.9% (56.0–69.4)	262/393; 66.7% (61.9–71.1)	73/335; 21.8% (17.7–26.5)	131/255; 51.4% (45.3–57.4)	0.276 (0.199–0.353)
**All ≥12 nodes**	462	282/462; 61.0% (56.5–65.4)	101/159; 63.5% (55.8–70.6)	181/303; 59.7% (54.1–65.1)	58/239; 24.3% (19.3–30.1)	122/223; 54.7% (48.2–61.1)	0.212 (0.125–0.298)
**pN0 requires ≥12 nodes**	500	305/500; 61.0% (56.7–65.2)	124/197; 62.9% (56.0–69.4)	181/303; 59.7% (54.1–65.1)	73/254; 28.7% (23.5–34.6)	122/246; 49.6% (43.4–55.8)	0.217 (0.134–0.300)
**Exclude eight records flagged for possible duplication**	582	381/582; 65.5% (61.5–69.2)	122/193; 63.2% (56.2–69.7)	259/389; 66.6% (61.8–71.1)	71/330; 21.5% (17.4–26.3)	130/252; 51.6% (45.4–57.7)	0.277 (0.196–0.352)
**Exclude two records with out-of-range nodal entries**	588	386/588; 65.6% (61.7–69.4)	124/196; 63.3% (56.3–69.7)	262/392; 66.8% (62.0–71.3)	72/334; 21.6% (17.5–26.3)	130/254; 51.2% (45.1–57.3)	0.280 (0.201–0.358)
**Exclude four records with staging-field anomalies**	586	386/586; 65.9% (61.9–69.6)	124/194; 63.9% (56.9–70.3)	262/392; 66.8% (62.0–71.3)	70/332; 21.1% (17.0–25.8)	130/254; 51.2% (45.1–57.3)	0.285 (0.208–0.361)
**Documentable protocol criteria**	571	377/571; 66.0% (62.0–69.8)	121/193; 62.7% (55.7–69.2)	256/378; 67.7% (62.9–72.2)	72/328; 22.0% (17.8–26.7)	122/243; 50.2% (44.0–56.4)	0.286 (0.208–0.362)

Proportion CIs are Wilson intervals. κ CIs were generated by 5,000 nonparametric bootstrap samples with fixed seeds. The pN0-specific analysis changes the negative reference set and should not be interpreted as a replacement prevalence estimate. CI, confidence interval; cN, CT-derived clinical nodal category; pN, pathological nodal category.

Table S3. Characteristics of patients with and without evaluable cN/pN.

Characteristic	Eligible base	cN/pN evaluable	cN and/or pN not evaluable
**Patients, n**	662	590	72
**Age, years, median (IQR)**	71.0 (65.0–77.0)	71.0 (65.0–77.0)	74.0 (65.8–78.2)
**Female sex, n/N (%)**	331/662 (50.0%)	300/590 (50.8%)	31/72 (43.1%)
**BMI, kg/m², median (IQR)**	26.7 (23.9–30.0)	26.6 (23.8–29.7)	27.1 (24.8–31.0)
**ASA III–V, n/N (%)**	227/650 (34.9%)	198/578 (34.3%)	29/72 (40.3%)
**Right/transverse colon, n/N (%)**	319/658 (48.5%)	288/586 (49.1%)	31/72 (43.1%)
**Left/rectosigmoid colon, n/N (%)**	339/658 (51.5%)	298/586 (50.9%)	41/72 (56.9%)
**cT0, n/N (%)**	4/631 (0.6%)	3/589 (0.5%)	1/42 (2.4%)
**cT1–2, n/N (%)**	185/631 (29.3%)	163/589 (27.7%)	22/42 (52.4%)
**cT3–4, n/N (%)**	442/631 (70.0%)	423/589 (71.8%)	19/42 (45.2%)
**Missing or unclassifiable cT, n/N (%)**	31/662 (4.7%)	1/590 (0.2%)	30/72 (41.7%)
**Centre A, n/N (%)**	78/662 (11.8%)	78/590 (13.2%)	0/72 (0.0%)
**Centre B, n/N (%)**	136/662 (20.5%)	100/590 (16.9%)	36/72 (50.0%)
**Centre C, n/N (%)**	58/662 (8.8%)	58/590 (9.8%)	0/72 (0.0%)
**Centre D, n/N (%)**	146/662 (22.1%)	111/590 (18.8%)	35/72 (48.6%)
**Centre E, n/N (%)**	244/662 (36.9%)	243/590 (41.2%)	1/72 (1.4%)
**Resection without neoadjuvant therapy, n/N (%)**	662/662 (100.0%)	590/590 (100.0%)	72/72 (100.0%)

The eligible base comprised nonmetastatic colon cancer resections without neoadjuvant therapy during the study period. Denominators vary according to data availability. Percentages for cT categories use patients with evaluable cT as the denominator and include cT0 as a distinct category. Percentages for missing or unclassifiable cT and centre distribution use the full size of each cohort. Resection without neoadjuvant therapy was an eligibility criterion for both groups. No formal hypothesis tests were used to compare included and excluded patients; these comparisons are descriptive and do not establish equivalence between groups. ASA, American Society of Anesthesiologists physical status; BMI, body mass index; cN, CT-derived clinical nodal category; cT, CT-derived clinical tumour category; IQR, interquartile range; pN, pathological nodal category.

Table S4. Diagnostic completeness and occult pN+ by anonymised centre.

Centre	Eligible base	cN not evaluable	pN not evaluable	Both	cN/pN complete	cN0	Occult pN+ among cN0 (95% CI)
**Centre A**	78	0	0	0	78/78 (100.0%)	65	8/65 (12.3%; 6.4–22.5)
**Centre B**	136	36	1	1	100/136 (73.5%)	50	19/50 (38.0%; 25.9–51.8)
**Centre C**	58	0	0	0	58/58 (100.0%)	40	12/40 (30.0%; 18.1–45.4)
**Centre D**	146	22	13	0	111/146 (76.0%)	73	12/73 (16.4%; 9.7–26.6)
**Centre E**	244	0	1	0	243/244 (99.6%)	107	22/107 (20.6%; 14.0–29.2)

cN and pN missingness categories overlap in the 'Both' column. CIs are Wilson intervals. Centre letters are anonymised and are not ordered by performance. CI, confidence interval; cN, CT-derived clinical nodal category; pN, pathological nodal category.

Table S5. Tumour location, corrected operative procedure, and surgical approach.

A. Exact clinical tumour location.

Location	Overall	cN0	cN+
**Cecum**	88/585 (15.0%)	54/331 (16.3%)	34/254 (13.4%)
**Ascending colon**	112/585 (19.1%)	45/331 (13.6%)	67/254 (26.4%)
**Hepatic flexure**	55/585 (9.4%)	34/331 (10.3%)	21/254 (8.3%)
**Transverse colon**	33/585 (5.6%)	21/331 (6.3%)	12/254 (4.7%)
**Splenic flexure**	23/585 (3.9%)	10/331 (3.0%)	13/254 (5.1%)

Location	Overall	cN0	cN+
**Descending colon**	36/585 (6.2%)	21/331 (6.3%)	15/254 (5.9%)
**Sigmoid colon**	178/585 (30.4%)	106/331 (32.0%)	72/254 (28.3%)
**Rectosigmoid**	60/585 (10.3%)	40/331 (12.1%)	20/254 (7.9%)

Exact subsite denominators were 585 overall, 331 for cN0, and 254 for cN+. One combined descending/sigmoid entry, one value outside the prespecified colon-subsite categories, and three missing values were not assigned to an exact subsite. The combined entry remained eligible for the left-sided analysis.

B. Operative procedure.

Procedure	Overall (N=590)	cN0 (n=335)	cN+ (n=255)
**Right hemicolectomy**	259/590 (43.9%)	137/335 (40.9%)	122/255 (47.8%)
**Transverse colectomy**	8/590 (1.4%)	6/335 (1.8%)	2/255 (0.8%)
**Left colectomy**	96/590 (16.3%)	47/335 (14.0%)	49/255 (19.2%)
**Extended right hemicolectomy**	13/590 (2.2%)	10/335 (3.0%)	3/255 (1.2%)
**Sigmoidectomy**	202/590 (34.2%)	129/335 (38.5%)	73/255 (28.6%)
**Hartmann procedure**	11/590 (1.9%)	6/335 (1.8%)	5/255 (2.0%)
**Pancolectomy**	1/590 (0.2%)	0/335 (0.0%)	1/255 (0.4%)

C. Surgical approach.

Approach	Overall (N=590)	cN0 (n=335)	cN+ (n=255)
**Open**	201/590 (34.1%)	93/335 (27.8%)	108/255 (42.4%)
**Laparoscopic**	204/590 (34.6%)	116/335 (34.6%)	88/255 (34.5%)
**Robotic**	185/590 (31.4%)	126/335 (37.6%)	59/255 (23.1%)

Procedure and approach are reported to describe operative case mix. They were not treated as determinants of CT performance. cN, CT-derived clinical nodal category.

Table S6. Post-hoc diagnostic estimates by clinical tumour side.

Tumour-side group	N	cN0/pN0; cN0/pN+; cN+/pN0; cN+/pN+	Accuracy (95% CI)	Sensitivity (95% CI)	Specificity (95% CI)	Occult pN+ among cN0 (95% CI)	pN0 among cN+ (95% CI)
**Right/transverse**	288	124/30/64/70	67.4% (61.7–72.5)	70.0% (60.4–78.1)	66.0% (58.9–72.4)	19.5% (14.0–26.4)	47.8% (39.5–56.2)
**Left/rectosigmoid**	298	136/42/67/53	63.4% (57.8–68.7)	55.8% (45.8–65.4)	67.0% (60.3–73.1)	23.6% (18.0–30.3)	55.8% (46.9–64.4)
**Right (cecum/ascending) [restricted]**	200	80/19/50/51	65.5% (58.7–71.7)	72.9% (61.5–81.9)	61.5% (53.0–69.5)	19.2% (12.6–28.0)	49.5% (40.0–59.1)
**Left (descending/sigmoid/ rectosigmoid) [restricted]**	275	129/39/62/45	63.3% (57.4–68.8)	53.6% (43.0–63.8)	67.5% (60.6–73.8)	23.2% (17.5–30.2)	57.9% (48.5–66.9)

Primary side groups classified caecum through transverse colon as right/transverse and splenic flexure through rectosigmoid as left/rectosigmoid; the single combined descending/sigmoid entry was retained as left-sided. The restricted comparison excluded hepatic-flexure, transverse-colon, and splenic-flexure tumours. Estimates are descriptive, Wilson 95% CIs overlap, and no between-side hypothesis test was prespecified. CI, confidence interval; cN, CT-derived clinical nodal category; pN, pathological nodal category.

https://doi.org/10.3390/xxxxx

Table S7. Characteristics of cN0 patients by pathological nodal status.

Characteristic	All cN0 (n=335)	cN0/pN0 (n=262)	cN0/pN+ (n=73)
Age, years, median (IQR)	72.0 (65.0–77.0)	72.0 (65.0–78.0)	71.0 (64.0–76.0)
Female sex, n/N (%)	165/335 (49.3%)	130/262 (49.6%)	35/73 (47.9%)
BMI, kg/m², median (IQR)	26.7 (24.2–30.0)	26.7 (24.2–29.7)	26.9 (24.5–31.0)
ASA III–IV, n/N (%)	116/331 (35.0%)	91/259 (35.1%)	25/72 (34.7%)
Right/transverse colon, n/N (%)	154/332 (46.4%)	124/260 (47.7%)	30/72 (41.7%)
cT3–4 among cT1–4, n/N (%)	181/331 (54.7%)	129/258 (50.0%)	52/73 (71.2%)
At least 12 nodes examined, n/N (%)	239/293 (81.6%)	181/227 (79.7%)	58/66 (87.9%)
Nodes examined, median (IQR)	16 (13–21)	16 (12–21)	17 (14–23)
R0 resection, n/N (%)	332/332 (100.0%)	260/260 (100.0%)	72/72 (100.0%)
Pathological tumour size, mm, median (IQR)	39 (25–50)	35 (25–45)	40 (30–50)
Grade 3, n/N (%)	19/329 (5.8%)	12/257 (4.7%)	7/72 (9.7%)

No hypothesis tests are shown because these comparisons support an exploratory association analysis rather than a prespecified causal contrast. Denominators vary because missing values were not imputed. The cT row uses the cT1–4 comparison population (258 pN0 and 73 pN+ patients); three cT0 cases and one missing or unclassifiable cT value are excluded. Thus, its denominator differs from the descriptive cT denominator in Table 1, which includes cT0. ASA, American Society of Anesthesiologists physical status; BMI, body mass index; cN, CT-derived clinical nodal category; IQR, interquartile range; pN, pathological nodal category; R0, microscopically margin-negative resection.

Table S8. Exploratory logistic models for occult pN+ among cN0 patients.

Model	N/events	Term	OR	95% CI	p value
Core: cT plus centre	331/73	cT3–4 vs cT1–2	3.10	1.71–5.62	<0.001
Core: cT plus centre	331/73	Centre B vs Centre A	5.96	2.27–15.64	<0.001
Core: cT plus centre	331/73	Centre C vs Centre A	5.67	1.96–16.42	0.001
Core: cT plus centre	331/73	Centre D vs Centre A	2.12	0.79–5.75	0.138
Core: cT plus centre	331/73	Centre E vs Centre A	2.52	1.03–6.17	0.044
Extended: cT, age, sex, side plus centre	328/72	cT3–4 vs cT1–2	3.19	1.71–5.94	<0.001
Extended: cT, age, sex, side plus centre	328/72	Age, per 10 years	0.84	0.65–1.09	0.200
Extended: cT, age, sex, side plus centre	328/72	Female vs male	0.89	0.51–1.56	0.693
Extended: cT, age, sex, side plus centre	328/72	Right/transverse vs left/rectosigmoid	1.04	0.57–1.92	0.895
Extended: cT, age, sex, side plus centre	328/72	Centre B vs Centre A	5.61	2.12–14.86	<0.001
Extended: cT, age, sex, side plus centre	328/72	Centre C vs Centre A	5.58	1.82–17.11	0.003
Extended: cT, age, sex, side plus centre	328/72	Centre D vs Centre A	2.00	0.74–5.45	0.173
Extended: cT, age, sex, side plus centre	328/72	Centre E vs Centre A	2.33	0.94–5.77	0.068
pN0 requires ≥12 nodes	251/73	cT3–4 vs cT1–2	2.38	1.26–4.48	0.007
Exclude cN0 records with staging-field anomalies	328/70	cT3–4 vs cT1–2	2.93	1.58–5.44	<0.001
Documentable protocol criteria	324/72	cT3–4 vs cT1–2	3.03	1.64–5.62	<0.001

Models comparing cT3–4 with cT1–2 excluded cT0 and missing or unclassifiable cT values. The core model included cT3–4 and centre fixed effects (six parameters including the intercept; 12.2 events per parameter). Centre A was the reference. The extended model contained nine parameters and 8.0 events per parameter and is reported as a sensitivity analysis. Models were exploratory association analyses and were not developed or validated for individual prediction. CI, confidence interval; cT, CT-derived clinical tumour category; OR, odds ratio.

Table S9. Panel A. Cross-classification of CT and pathological T category.

CT category	pT0	pT1	pT2	pT3	pT4	Total
**cT0**	2	1	0	0	0	3
**cT1**	9	23	7	4	0	43
**cT2**	18	9	65	39	4	135
**cT3**	1	1	48	262	21	333
**cT4**	0	1	6	62	35	104
**Total**	30	35	126	367	60	618

N = 618; exact agreement 387/618 (62.6%); CT under-classification 76/618 (12.3%); CT over-classification 155/618 (25.1%); linear-weighted κ=0.502 (95% CI, 0.448–0.554). All observations coded as T0 are grouped in the cT0 row or pT0 column, respectively; T0 is retained as a distinct category from T1–T4. The 3 cT0 and 30 pT0 observations overlap in 2 cT0/pT0 pairs. One additional cT0 case without evaluable pT is excluded from this paired analysis. cT0 is included in descriptive denominators (Table 1 and Table S3) but excluded from the cT1–2 versus cT3–4 comparison (Tables S7 and S8). All T4 subcategories and database-specific variants were combined as T4. CI, confidence interval; CT, computed tomography; pT, pathological T category.

Table S9. Panel B. Availability of cT and pT according to inclusion in the primary nodal analysis.

T-stage data availability	Otherwise eligible patients (N=662)	Included in the nodal analysis (N=590)	Excluded from the nodal analysis (N=72)
**Both cT and pT evaluable**	618	589	29
**cT non-evaluable; pT evaluable**	29	1	28
**cT evaluable; pT non-evaluable**	13	0	13
**Neither cT nor pT evaluable**	2	0	2
**Total**	662	590	72

Values are patient counts. Non-evaluable denotes missing or unclassifiable values under the study's T-category harmonisation rules. The paired T-stage analysis required evaluable cT and pT, irrespective of cN/pN completeness. T0 was retained as a distinct evaluable category.

Table S10. Nodal-yield adequacy across cN/pN cells.

cN	pN	N	Nodes known	<12 nodes	≥12 nodes	Nodes missing
**cN0**	pN0	262	227	46	181	35
**cN+**	pN0	131	127	5	122	4
**cN0**	pN+	73	66	8	58	7
**cN+**	pN+	124	112	11	101	12

The ≥12-node threshold is a quality benchmark for confidence in a negative pathological nodal assessment; it is not a guarantee that every metastasis was detected. cN, CT-derived clinical nodal category; pN, pathological nodal category.
